# Impaired interferon‐α expression in plasmacytoid dendritic cells in asthma

**DOI:** 10.1002/iid3.376

**Published:** 2020-11-25

**Authors:** Ting‐Yu Lin, Chun‐Yu Lo, Kuo‐Chien Tsao, Po‐Jui Chang, Chih‐His Scott Kuo, Yu‐Lun Lo, Shu‐Min Lin, Meng‐Heng Hsieh, Tsai‐Yu Wang, Ping‐Chih Hsu, Horng‐Chyuan Lin

**Affiliations:** ^1^ Department of Thoracic Medicine Chang Gung Memorial Hospital Taipei Taiwan; ^2^ Department of Medicine, School of Medicine Chang Gung University Taipei Taiwan; ^3^ Department of Laboratory Medicine Lin‐Kou Chang Gung Memorial Hospital Taoyuan Taiwan

**Keywords:** asthma, dendritic cells, rhinovirus, toll‐like receptor 7

## Abstract

**Background:**

Toll‐like receptor (TLR)‐7‐associated rhinovirus (RV) activation is involved in the pathogenesis of asthma. Plasmacytoid dendritic cells (pDCs) are the main interferon‐α‐producing cells against viruses.

**Objective:**

To determine whether asthmatic patients and control subjects differ in terms of interferon‐α expression in pDCs under TLR‐7 or RV stimulation.

**Methods:**

pDCs were identified in BDCA‐2+ and HLA‐DR+ peripheral blood mononuclear cells. Interferon‐α expression of pDCs was analyzed after TLR‐7 stimulation with or without interleukin 4 (IL‐4)/IL‐13 pretreatment. Interferon‐α expression was also analyzed after RV stimulation over periods of 24, 48, or 96 h with or without IL‐4 pretreatment. RV detection and molecular typing were assayed from throat swabs.

**Results:**

Following TLR‐7 stimulation, the expression of intracellular interferon‐α was higher in the pDCs of normal subjects than those of asthmatic patients; however, pretreatment with IL‐4 was shown to reduce this effect. After 48‐ and 96‐h RV stimulation, we observed a notable increase in the production of interferon‐α of pDCs in normal subjects but not in asthmatic patients. Baseline interferon‐α expression in pDCs and the incidence of asthma exacerbation to emergency was higher among the 13% of patients identified as rhinovirus+ than among their RV counterparts.

**Conclusion:**

Our study discovered the response to TLR‐7 stimulation in pDCs was compromised and the sustainability of interferon‐α expression to RV stimulation was reduced in pDCs of asthmatic patients, which provide further evidence of defective innate response and subspeciality to RV infection in asthma. The high exacerbation history founded in RV+ patients agrees with these findings. Further research is required for the modulatory effect of IL‐4 on TLR‐7 stimulated pDCs.

## Graphical abstract

Our study discovered the response to TLR‐7 stimulation in pDCs was compromised and the sustainability of interferon‐a expression to RV stimulation was reduced in pDCs of asthmatic patients, which provide further evidence of defective innate response and subspeciality to RV infection in asthma. The high exacerbation history founded in RV+ patients agrees with these findings.

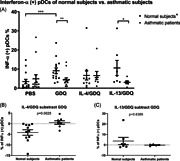

## 1 BACKGROUND

Virus infection is an important risk factor for asthma attacks.^1^, ^2^, ^3^ In fact, 60% of asthma exacerbations among adults are specifically associated with rhinovirus (RV) infection.^4^, ^5^ Researchers have clearly demonstrated the adverse effects of RV on the pathogenesis of asthma, including airway hyperresponsiveness, airway obstruction, impaired epithelial barriers, and type 2 inflammation.^6^, ^7^, ^8^, ^9^

RV is a single‐stranded RNA virus with three species identified by sequence homology as RV‐A, B, and C. Intercellular adhesion molecule‐1 (ICAM‐1) is the key epithelial cell receptor for RV‐A and B. Under RV infection, interferon production (type I: α and β, and type 3: λ) is triggered when endosomal pattern recognition receptors (PRRs) are activated by viral genomic material. Note that interferon is the main antiviral cytokine involved in innate immunity. PRRs include toll‐like receptor (TLR) 7 or 8 (activated by the single‐stranded RV genome) and TLR3 (activated by the double‐stranded RNA formed during viral replication).^10^

Asthmatic patients are more susceptible than non‐asthmatic subjects to viral infection, due presumably to the upregulation of ICAM‐1 in the epithelium,^11^ decreased ICAM‐1 solubility,^3^ and impaired interferon response to viruses in the epithelium, alveolar macrophages, and peripheral blood mononuclear cells (PBMCs), which can compromise viral clearance.^12^, ^13^, ^14^, ^15^ Among PBMCs, plasmacytoid dendritic cells (pDCs) are the most prolific in terms of type I interferon production following viral recognition relying on the expression of TLR‐7 or TLR‐9.^16^, ^17^ Researchers have shown that add‐on anti‐immunoglobulin E (IgE) therapy can reduce the incidence of RV‐associated exacerbation in asthmatic children.^18^ Anti‐IgE therapy is seen as one possible approach to restoring the innate response to RV infection, by reducing FcϵRIα cross‐linking on pDCs through a decrease in the circulation of IgE and downregulation of FcϵRI on pDCs.^19^, ^20^ Studies demonstrated that the expression of TLR‐7 in the bronchial biopsy of patients with eosinophilic asthma or in the alveolar macrophage of severe asthma was defective^21^, ^22^; however, there is a lack of evidence indicating abnormal TLR‐7 function in the pDCs of asthma patients. Our objective in this study was to determine whether the innate response of pDCs to TLR‐7 and RV is defective in patients with asthma. This was achieved by comparing the expression of interferon‐α in pDCs isolated from the PBMCs of asthmatic patients and normal subjects under stimulation from a TLR‐7 agonist and RV.

## 2 METHODS

### 2.1 Subjects and blood sampling

This study recruited asthmatic patients with a history of one or more of the following: (1) Greater than 12% improvement in forced expiratory volume in 1 s (FEV1) following the use of a bronchodilator; (2) PC20 of methacholine test less than 8 mg/ml; (3) diurnal variation of greater than 20% in peak expiratory flow; (4) increase in FEV1 by greater than 12% and 200 ml or diurnal variation in peak expiratory flow by greater than 20% from baseline after four weeks of treatment; (5) patients with fixed airway obstruction who responded positively to asthma medication. Control level of asthma was defined in accordance with guidelines of the current global initiative for asthma. Subjects were non‐smokers and clinically free from respiratory or systemic infection during the previous 2 months and provided written informed consent from the Chang Gung Medical Foundation Institutional Review Board (No. 201601945A3).

### 2.2 Preparation of PBMCs

Samples of peripheral blood (50 ml) were obtained from patients and normal subjects. PBMCs were isolated from whole blood by using Ficoll‐Paque density gradient centrifugation (GE Healthcare, Bio‐science AB) under 805 g at 20°C for 20 min. PBMCs were cultured in Roswell Park Memorial Institute (RPMI) 1640 (Gibco Life Technologies) with interleukin 3 (IL‐3) (10 ng/ml).^23^ Note that 30% of the volume comprised the patient's own serum to promote pDC survival. A portion of the PBMCs were stained using surface markers to quantify the porportion of pDCs in the PBMCs. The remaining PBMCs underwent stimulation using a TLR‐7 agonist or RV.

### 2.3 RV detection and molecular typing

Throat swabs were placed immediately after collection into viral transport medium. Viral RNA was extracted using LabTurbo Viral DNA/RNA Extraction Kit (TaiGen Biotechnology Inc.) in accordance with the manufacturer's instructions. Reverse transcription was performed using SuperScript III First‐Strand Synthesis System (Life Technologies) in accordance with the manufacturer's protocol. The VP4/VP2 amplicon of the RV strains was amplified using semi‐nested polymerase chain reaction (PCR) with the RV forward (F484‐5′‐CGGCCCCTGAATGYGGCTAA‐3′ and F587sn‐5′‐CTACTTTGGGTGTCCGTGTTTC‐3′) and reverse primers (R1126‐5′‐ATCHGGHARYTTCCAMCACCA‐3′) as previously described.^24^ The RV species of each strain was identified via direct sequencing of VP4/VP2 PCR products. The PCR products were purified from agarose gel using a gel extraction kit (QIAGEN, Germany) in accordance with the manufacturer's specifications. The purified DNA served as templates for chain termination reactions using the ABI 3730 XL DNA Analyzer (Applied Biosystem Inc.). All of the amplification products were sequenced bidirectionally to confirm amplification specificity and virus typing via phylogenetic analysis.

### 2.4 Preparation of RV‐A16

RV‐A16 was purchased from ATCC (ATCC VR‐283). H1‐HeLa cells were obtained from ATCC (CRL‐1958) and maintained in minimum essential medium (MEM) (Gibco, Thermo Fisher Scientific) supplemented with 10% fetal bovine serum (FBS), 1% antibiotic‐antimycotic (Thermo Fisher Scientific), and 200 mM *l*‐Glutamine with incubation at 37°C in an incubator under 5% CO_2_. Viral stocks were generated by infecting monolayer cultures of H1‐HeLa (ATCC CRL‐1958) cells in MEM medium supplemented with 2% FBS and incubated at 33°C under 5% CO_2_, and then observed for cytopathic effects. H1‐HeLa cells were harvested, lysed by freezing and thawing, and then pelleted via centrifugation. The supernatants containing RV‐A16 were transferred to fresh tubes as virus stocks and stored at −70°C. Viral titers were determined by plaque assay. Briefly, confluent H1‐HeLa cell monolayers in six‐well plates were washed twice using phosphate‐buffered saline (PBS), whereupon 10‐fold serial dilutions of the virus (0.5 ml) were adsorbed onto cells at 33°Cover a period of one hour. Any unadsorbed viruses were removed by washing with PBS. The cells were then covered with an overlay of MEM containing 2% FBS and 0.3% purified agarose. After 72‐h incubation at 33°C, the cells were fixed using 10% formalin for 2 h. After removing the formalin, the cells were stained using 1% crystal violet, whereupon viral titers of plaque‐forming units per milliliter were determined by visible plaques (Figure S1).^25^

### 2.5 Identification of pDCs in PBMCs and receptor expression using flow cytometry

**Figure 1:**
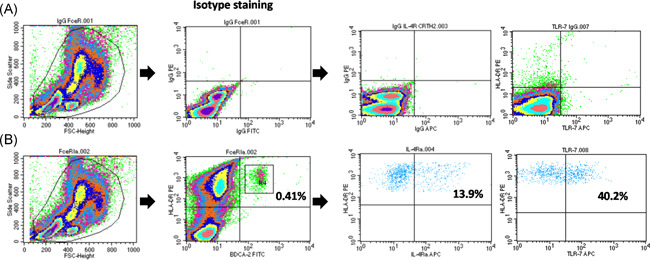
Strategy for identifying plasmacytoid dendritic cells (pDCs) in peripheral blood mononuclear cells (PBMCs). (A) Isotype staining. (B) Identification of pDCs based on double expression of HLA‐DR‐PE and BDCA2‐FITC populations in PBMCs (left two panels) and the expression of interleukin 4 receptor α (IL‐4Rα) or toll‐like receptor 7 (TLR‐7) by APC (right three panels). APC, allophycocyanin

PBMCs (1.5 × 10^6^ cells/1.5 ml) were stained using two markers to identify pDCs: BDCA‐2‐FITC (MACS Miltenyi Biotec) and HLADR‐PE (MACS Miltenyi Biotec). The PBMCs were then incubated with IL4‐Ra‐APC (R&D Systems) or TLR‐7‐APC (Thermo Fisher Scientific) (Figure 1). The addition of fluorescence‐conjugated antibodies for flow cytometry was conducted in accordance was the manufacturer's protocol. TLR‐7 staining involved staining with BDCA‐2 and HLA‐DR followed by the fixing of PBMCs with BD FACS Permeabilizing Solution 2 (BD Biosciences) for 20 min and then resuspension with RPMI 1640 for further APC staining. A total of 3 × 10^5^ cells were acquired and analyzed on a BD FACScan flow cytometer (BD Biosciences) using CellQuest software.

### 2.6 Effects of TLR‐7 stimulation on intracellular interferon‐α expression of pDCs

**Figure 2:**
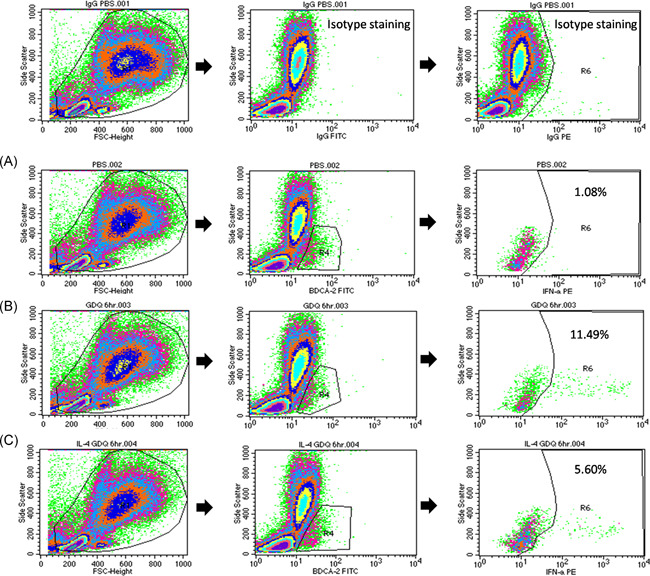
Gating strategy for the intracellular interferon‐α staining of pDCs under TLR‐7 stimulation. (A) Compared to control conditions using phosphate‐buffered saline (PBS), (B) TLR‐7 stimulation increased the portion of pDCs identified as positive for interferon‐α‐PE to 11.9%, (C) the effects of which were reduced to 5.6% by IL‐4 pretreatment. Left panel: Whole cell population. Middle panel: BDCA‐2+ cell population. Right panel: Interferon‐α + staining gating from BDCA2+ cell population. IL‐4, interleukin 4; pDC, plasmacytoid dendritic cell; TRL‐7, toll‐like receptor 7

We also evaluated effects of TLR‐7 on the production of interferon‐α from pDCs under the Th2 immune milieu in asthmatic subjects and non‐asthmatic normal controls *ex vivo*. This was achieved by isolating PBMCs, pretreating them with IL‐4 (4 ng/ml), IL‐13 (10 ng/ml), or PBS for 1 h, followed by TLR7 stimulation via the TLR7 agonist Gardiquimod (InvivoGen) (0.5ug/ml) for 6 h as well as Brefeldin‐A (Thermo Fisher Scientific) for the last 3 h. Cells then underwent staining with BDCA‐2‐FITC to estimate pDCs surface marker expression and interferon‐α‐PE staining (MACS Miltenyi Biotec) after fixing with permeabilizing solution for intracellular antigen detection (Figure 2).

### 2.7 Effects of RV stimulation on intracellular interferon‐α expression in pDCs

We evaluated the effects of RV stimulation on the ex vivo production of interferon‐α in pDCs from asthmatic subjects and non‐asthmatic normal controls. This involved treating isolated PBMCs with Gardiquimod (0.5 µg/ml) for 6 h as well as Brefeldin‐A for the last 3 h, PBS for 6 h as well as Breldin‐A for the last 3 h, or RV‐A16 (multiplicity of infection [MOI] = 1) for 24 and 48 h as well as Brefeldin‐A for the last 3 h. Cells then underwent staining with BDCA‐2‐FITC to estimate pDCs surface marker expression and also stained with interferon‐α‐PE for intracellular antigen detection (Figure S2 in Supplemental Materials). One additional experiment was performed involving extended RV stimulation with or without pretreatment of IL‐4 for 96 h.

#### 2.7.1 Enzyme‐linked immunosorbent assay

Supernatants of PBMC cultures under TLR‐7 agonist and RV stimulation were assayed for interferon‐α cytokine using an nzyme‐linked immunosorbent assay (ELISA) kit (R&D Systems). Note that the level of interferon‐α obtained here represented the results under the stopping protein transport by Breldin‐A for the last 3 h.

### 2.8 Statistical analysis

Data were expressed as mean ± *SD*. An unpaired two‐tailed Student's *t* test was used for single comparisons. The *χ* ^2^ Test was used to compare categorical data. Comparison of all stimulation within normal subjects or asthmatic patients was done by repeated measures analysis of variance (ANOVA) with Dunnet post hoc test. Comparison of the same stimulation between normal subjects or asthmatic patients was done by Mann–Whitney test. A one‐way ANOVA with Dunnett's test was used to compare the effects of PBS treatment. Probability values of less than .05 were considered significant.

The data that support the findings of this study are available from the corresponding author upon reasonable request.

## 3 RESULTS

**Figure 3:**
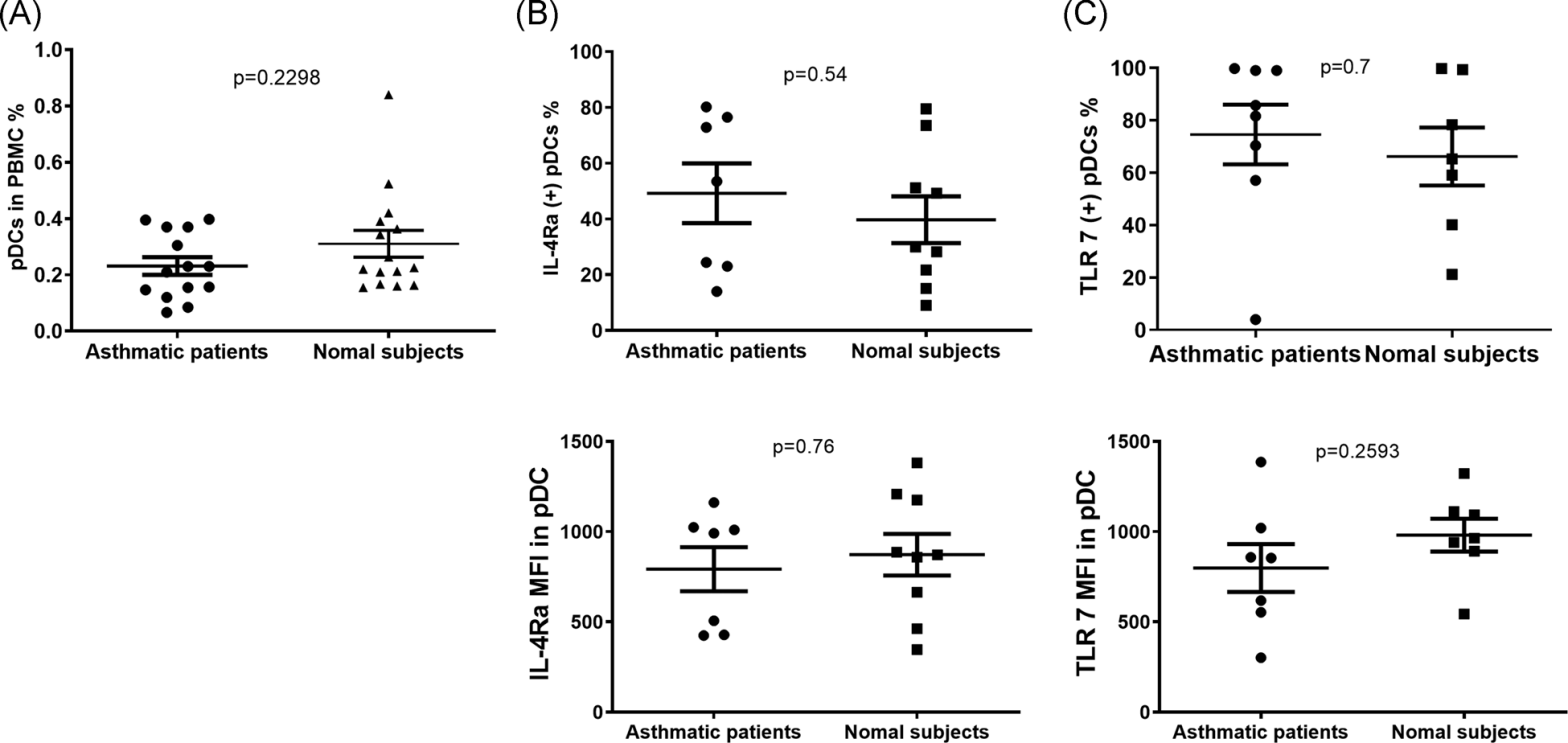
The proportion of pDCs in (A) PBMCs (asthma, *n* = 14; normal subjects, *n* = 15), (B) expression of IL‐4Rα (asthma, *n* = 7; normal subjects, *n* = 9), and (C) TLR‐7 (asthma, *n* = 7; normal subjects, *n* = 8) by % of pDCs and MFI in asthmatic subjects and normal subjects. IL‐4Rα, interleukin 4 receptor α; MFI, mean fluorescence intensity; pDC, plasmacytoid dendritic cell; PBMC, peripheral blood mononuclear cell; TRL‐7, toll‐like receptor 7

Asthmatic patients (*n* = 26) and normal subjects (*n* = 26) were recruited to provide blood samples (Table S1 in the Supplemental Materials). Among the asthmatic patients, 23 agreed to provide throat swabs for RV identification. The proportion of pDCs in PBMCs was 0.23% ± 0.12% and 0.31% ± 0.18% in asthmatic patients (all severities) and normal subjects, respectively (Figure 3A). No differences were observed between asthmatic patients and normal subjects in terms of hIL‐4Ra or TLR‐7 expression in pDCs (Figure 3B,C).

### 3.1 Production of interferon‐α in PBMCs and pDCs following TLR‐7 agonist stimulation in type 2 inflammation milieu

We analyzed the level of interferon‐α in the supernatant from the culture of PBMCs as well as the intracellular expression in pDCs after 6‐h stimulation with the TLR‐7 agonist Gardiquimod, as well as with or without IL‐4 or IL‐13 pretreatment.

**Figure 4:**
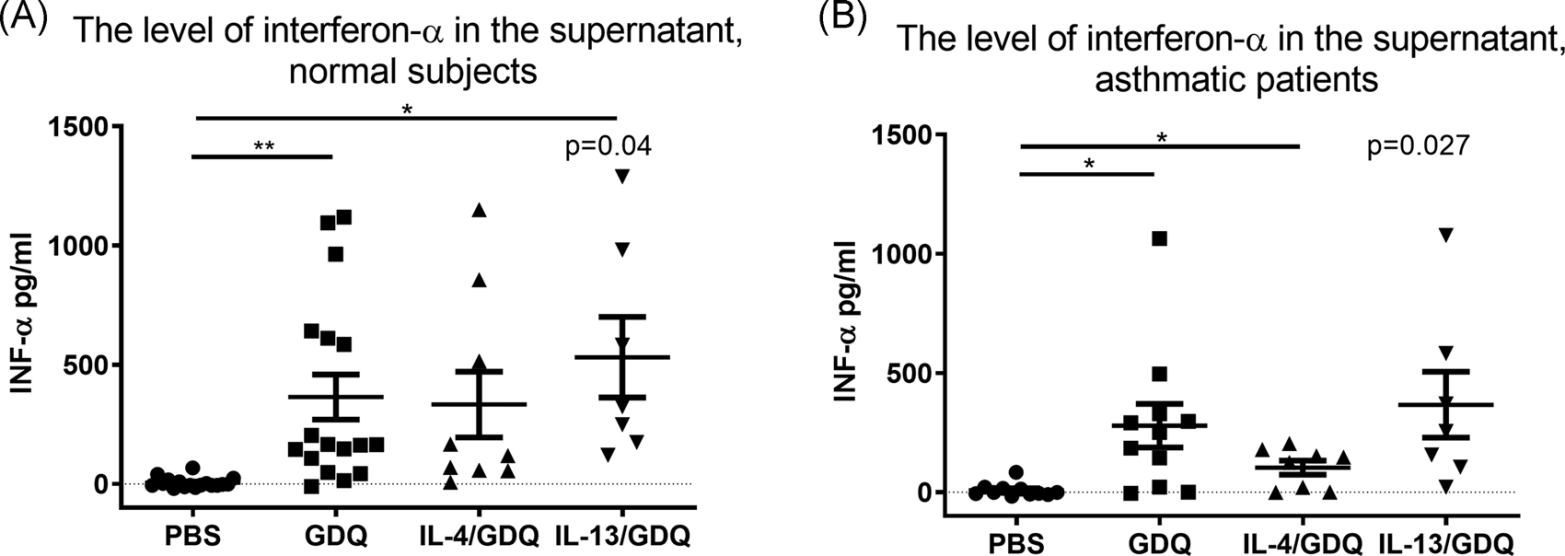
Interferon‐α production in PBMC cultures under TLR‐7 stimulation. Gardiquimod (GDQ) with IL‐4 or IL‐13 pretreatment. (A) pDCs of normal subjects (*n* = 17) and (B) asthmatic patients (*n* = 11). INF‐α, interferon α; IL, interleukin; PBS, phosphate‐buffered saline; pDC, plasmacytoid dendritic cell; PBMC, peripheral blood mononuclear cell; TRL‐7, toll‐like receptor 7

Following Gardiquimod stimulation, normal subjects and asthmatic patients both presented a significant increase in the level of interferon‐α (Figure 4), and pretreatment with IL‐13 was shown not to affect these results. Pretreatment with IL‐4 reduced the effect of Gardiquimod on interferon‐α production to levels that were not significantly distinct from the control condition with PBS. Furthermore, no differences were observed between asthmatic patients and normal subjects in terms of the effects of Gardiquimod and pretreatment with IL‐4 or IL‐13 (Figure S3 in the Supplemental Materials).

**Figure 5:**
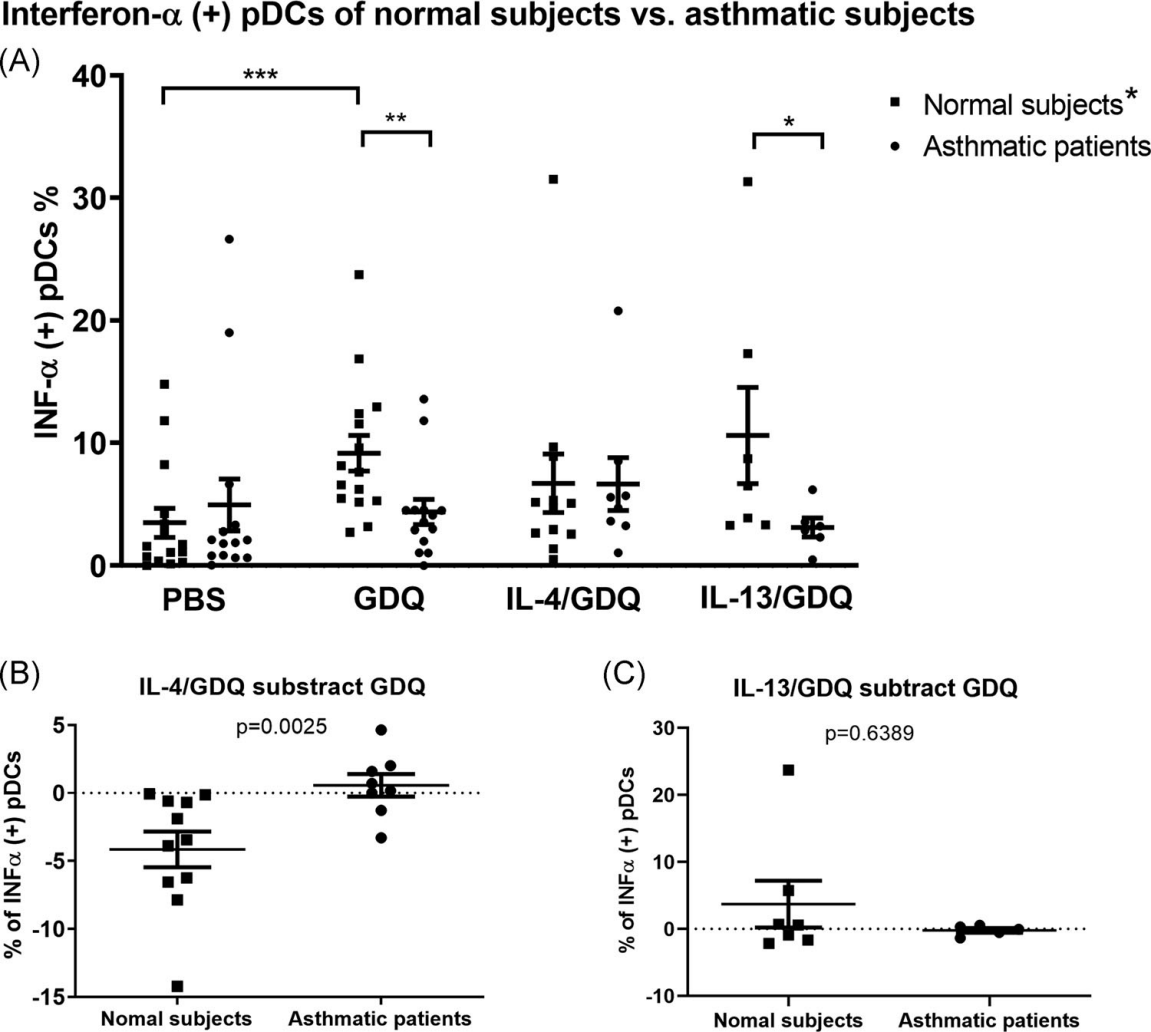
(A) Intracellular interferon‐α expression of pDCs after TLR‐7 agonist, GDQ, stimulation in the type 2 inflammation milieu in normal subjects (*n* = 14) and asthmatic patients (*n* = 15). Significant increasing interferon‐α (+) pDCs after GDQ stimulation compared to PBS in normal subjects; Significant more interferon‐α (+) pDCs after GDQ stimulation of normal subjects than that of asthmatic patients and significant more interferon‐α (+) pDCs after GDQ stimulation with IL‐13 pretreatment of normal subjects than that of asthmatic patients. (B) Difference of intracellular interferon‐α expression in the pDCs after GDQ without and with IL‐4 pretreatment of normal subjects and asthmatic patients. The Decreasing interferon‐α (+) pDCs after GDQ stimulation with IL‐4 in normal subjects was significant more than that of asthmatic patients, (C) Difference of intracellular interferon‐α expression in the pDCs after GDQ without and with IL‐13 pretreatment of normal subjects and asthmatic patients. Comparison of all stimulation within normal subjects or asthmatic patients was done by Repeated measures ANOVA with Dunnet post hoc test. Comparison of the same stimulation between normal subjects and asthmatic patients was done by Mann–Whitney test. ANOVA, analysis of variance; GDQ, Gardiquimod; INF‐α, interferon α; IL, interleukin; PBS, phosphate‐buffered saline; pDC, plasmacytoid dendritic cell; TRL‐7, toll‐like receptor 7. *p < .05, **p < .01, and ***p < .001

We observed higher intracellular interferon‐α expression levels in the pDCs of normal subjects after Gardiquimod stimulation, compared to PBS control (Figure 5A). The expression of intracellular‐α did not increase after Gardiquimod stimulation in asthmatic patients. There was no difference between the groups in terms of PBS control. After pretreatment with IL‐4, the effect of Gardiquimod on the level of intracellular interferon‐α in pDCs was no significant different from PBS in normal subjects as well as asthmatic patients. Note that the difference in intracellular interferon‐α levels following Gardiquimod treatment with and without IL‐4 pretreatment was more pronounced in normal subjects than in asthmatic patients (Figure 5B). Pretreatment with IL‐13 did not have this effect (Figure 5C). In terms of disease severity and status of atopy, no between‐group differences were observed in the expression of intracellular interferon‐α in pDCs after Gardiquimod stimulation (Figure S4 in the Supplemental Materials).

### 3.2 Production of interferon‐α by PBMCs and the intracellular expression of interferon‐α in pDCs following RV stimulation

**Figure 6:**
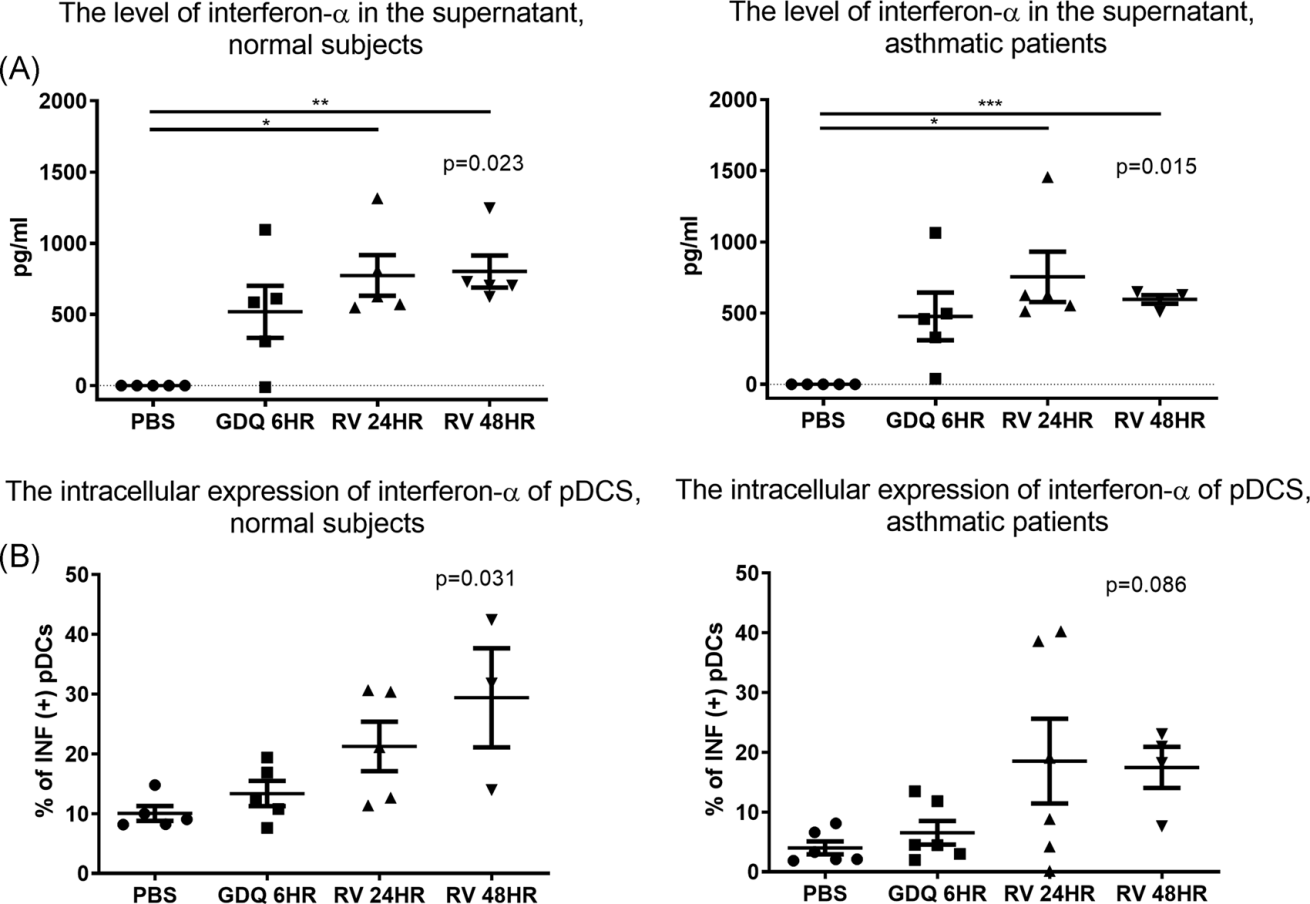
Production of INF‐α in the PBMCs of (A) normal subjects and (B) asthmatic patients; and the expression of intracellular interferon‐α in the pDCs of (C) normal subjects and (D) asthmatic patients after rhinovirus (RV) stimulation. (normal subjects, *n* = 5; asthma, *n* = 6). GDQ, Gardiquimod; IL, interleukin; INF‐α, interferon α; PBMC, peripheral blood mononuclear cell; PBS, phosphate‐buffered saline; pDC, plasmacytoid dendritic cell

We also investigated the response of pDCs to RV stimulation. In a preliminary test, the expression of ICAM‐1 in pDCs exceeded 95% (Figure S5 in Supplemental Materials). Our objective in the first experiment was to determine whether RV‐A16 (MOI 1) stimulation would induce interferon‐α production in pDCs. Note that samples undergoing 6‐h Gardiquimod stimulation were used as a positive control. We observed a significant increase in the level of interferon‐α in the supernatant from PBMC cultures following RV‐A16 stimulation (24‐ and 48‐h) in normal subjects as well as asthmatic patients (Figure 6A). Note that the normal subjects did not present a significant increase in the expression of intracellular interferon‐α by pDCs until 48 h (Figure 6B). After 48‐h RV‐A16 stimulation, the level of interferon‐α was higher in the supernatant of PBMCs cultures from normal subjects than from asthmatic patients (*p* = .065, Figure S6C in the Supplemental Materials). Thus, we sought to determine whether extending the duration of RV stimulation with IL‐4 pretreatment would have the same effects on the pDCs in asthmatic patients and normal subjects.

### 3.3 Production of interferon‐α in PBMCs and expression of intracellular interferon‐α in pDCs after 96‐hour RV stimulation in type 2 inflammation milieu

**Figure 7:**
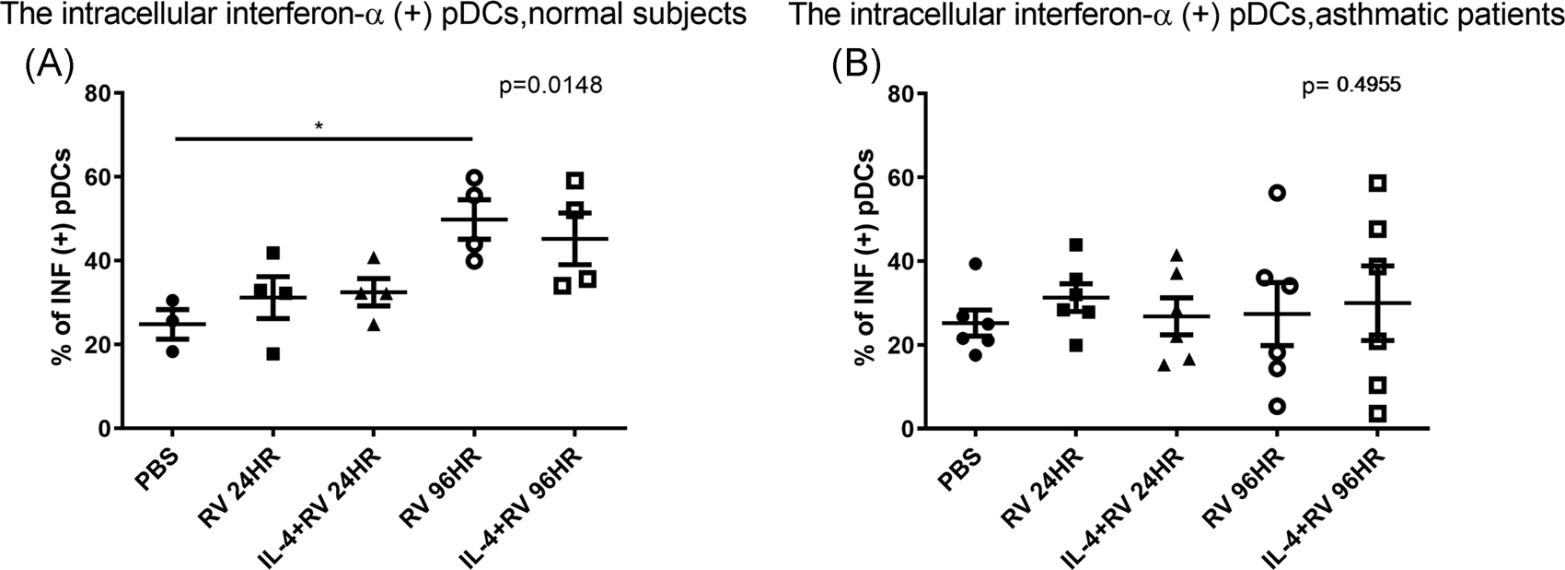
Expression of intracellular INF‐α in pDCs after 24‐ and 96‐h RV stimulation with or without IL‐4 pretreatment. (A) Normal subjects (*n* = 4) and (B) asthmatic patients (*n* = 6). IL, interleukin; INF, interferon; PBS, phosphate‐buffered saline; pDC, plasmacytoid dendritic cell; RV, rhinovirus

PBMCs were treated with RV‐A16 (MOI 1) for 24 or 96 h with or without IL‐4 pretreatment. Similar to the previous experiment, 24‐h RV stimulation did not significantly increase interferon‐α production in pDCs in either group. As shown in Figure 7A, increasing RV incubation to 96 h significantly increased intracellular interferon‐α expression in normal subjects; however, this effect was not significant in samples from normal subjects after undergoing pretreatment with IL‐4. Note that there was no effect in samples from asthmatic patients. In both normal subjects and asthmatic patients, there was a significant increase in interferon‐α levels in the supernatant of PBMC cultures after RV stimulation (Figure S7 in the Supplemental Materials).

Moreover, there was no correlation between intracellular interferon‐α expression in pDCs and asthma severity, pulmonary function, or the atopic status of asthmatic patients (Figure S8 in the Supplemental Materials).

### 3.4 RV molecular typing and quantification in asthmatic patients

RV was identified from the throat swabs of 3 of the 23 patients (RV+ patients, Table 1). All RV+ patients had received emergency treatment for asthma exacerbation in the previous year. Note that the incidence of emergency care was significantly higher among RV+ than among RV− patients (100% vs. 10%, *p* = .006). The expression of interferon‐α in pDCs treated with PBS was significantly higher among RV + patients than among RV− patients (Figure S9A in Supplemental Materials). After TLR‐7 stimulation (with or without IL‐4 pretreatment), there was no difference between the two groups in terms of interferon‐α expression in pDCs (Figure S9B and S9C).

**Table 1:** Patient characteristics, pulmonary function, and asthma severity among patients with RV (+) based on throat swabs

**Case**	**HRV 5**′**‐UTR real‐time PCR copies**	**HRV genotypes**	**Age**	**Gender**	**IgE**	**Atopy**	**FEV1% of predict**	**FEV1/FVC, %**	**Airway reversibility**	**Asthma severity**
1	<50 copies	HRV‐C27	68	Female	20	no	65	83.1	PC20: 0.79	Severe asthma
2	<50 copies	HRV‐A8	67	Male	217	Mite	90	77.2	post BD FEV1 change: 17%	Soderate asthma
3	279	HRV‐A44	56	Female	3.32	no	45	71	post BD FEV1 change: 16%	Severe asthma

Abbreviations: FEV1, forced expiratory volume in 1 s; FVC, forced vital capacity; HRV, human rhinovirus; IgE, immunoglobulin E; PCR, polymerase chain reaction; RV, rhinovirus; 5′‐UTR, 5′‐untranslated region.

## 4 DISCUSSION

We found that after TLR‐7 stimulation, the expression of intracellular interferon‐α in pDCs was lower among asthmatic patients than among normal subjects. IL‐4 pretreatment could reduce the expression of interferon‐α in the pDCs of normal subjects under TLR‐7 stimulation. Following RV stimulation, there was an increase in interferon‐α levels in normal subjects from 48 to 96 h; however, no such increase was observed in asthmatic patients. These results indicate that under TLR‐7 stimulation, the production of interferon‐α in pDCs is defective in asthmatic patients. They also indicate that in normal subjects, IL‐4 can compromise the TLR‐7 stimulated production of interferon‐α in pDCs. During RV infection, the expression of interferon‐α in pDCs persisted longer in normal subjects than in asthmatic patients. We found that after TLR‐7 or RV stimulation, there was an increase in interferon‐α levels in the supernatant of PBMC cultures from normal subjects as well as asthmatic patients. Baseline interferon‐α expression in pDCs and the incidence of asthma exacerbation to emergency was higher among the 13% of patients identified as RV+ than among their RV‐ counterparts.

Our results demonstrated the TLR‐7 response of pDCs was compromised and the sustainability of interferon‐α expression to RV stimulation was reduced in asthma, which discovered potential mechanisms of reduced innate response for viral clearance in asthma and provided additional evidence of previous works.^4^, ^26^, ^27^ Two studies have showed the illnesses associated with viruses have greater duration and severity in asthmatic patients.^4^, ^26^ Zambrano et al.^27^ further showed asthmatic patients had greater RV associated respiratory symptoms up to 21 days compared to the control subjects. Therefore, good personal hygiene to keep viral infection away are important part of asthma care.

pDCs play a key role in mediating innate immune responses to viral infection. Previous studies have reported on the genetic variability and functional defects associated with TLR‐7 in cases of asthma.^22^ Our results provide direct evidence that TLR‐7 stimulated interferon‐α production in pDCs is compromised in asthmatic patients. Note that our findings are in line with those reported by Bratke et al.^28^ Recent studies have shown that the TLR‐7 signal pathway is fine‐tuned by regulatory receptors expressed by pDCs.^29^, ^30^ Furthermore, the cross‐linking of FcϵRIα and regulatory receptors (e.g., BDCA2 and ILT7) forms complexes, which transduce immunoreceptor tyrosine‐based activation motif (ITAM) signaling in the negative regulation of TLR7/9‐induced type I interferon production.^31^, ^32^ Other receptors (e.g., NKp44, Siglec‐H, and pDC‐TRDM) form complexes with DNAX activation protein 12 to activate ITAM signaling.^33^ Elevated expression of FcϵRIα in pDCs has been observed in cases of acute asthma exacerbation; however, researchers have yet to elucidate the function of other ITAM‐associated regulatory receptors. Besides, mitochondrial antiviral‐signaling protein–etinoic acid‐inducible gene I (MAVS‐RIGI) pathway which is also important for interferon‐I production in virally infected cells, including pDCs.^34^ This raises the question as to whether there are any phenotypic difference between the pDCs in asthma patients and normal patients.^35^ Further research will be required to identify the role played by theese regulatory receptors of ITAM or MAVS‐RIGI pathway of pDCs in the pathogenesis of asthma.

It has been shown that IgE cross‐linking on pDCs and aberrant expression of microRNA in alveolar macrophages compromised TLR‐7 expression in asthma.^22^, ^36^ One novel finding in the current study was the fact that IL‐4 can undermine the effects of TLR‐7 stimulation on interferon‐α production in the pDCs of normal subjects, as indicated by a reduction in interferon‐α production in PBMC cultures under TLR‐7 stimulation following pretreatment with IL‐4 (Figure 5). It has previously been demonstrated that IL‐4 can reduce tumor necrosis factor‐α or IL‐1β production in monocytes or endothelial cells.^37^, ^38^ Further research will be required to identify the mechanism by which IL‐4 suppresses pDCs (e.g., STAT6 signaling).^38^ Note that these suppression effects were not observed in asthmatic patients, due perhaps to an insignificant increase in interferon‐α levels in the pDCs of asthmatic patients after TLR‐7 stimulation. IL‐4 did not affect the interferon‐α expression in pDCs by RV stimulation (Figure 7), possible due to the prolong duration of RV stimulation (24 and 96 h).

Deficient interferon production in response to RV stimulation in asthma has been studied in different cell types. Some studies showed the defective interferon‐λ or β production of asthmatic bronchial epithelial cells infected with RV ex vivo^15^, ^39^, ^40^ but the study of Sykes et al.^41^ did not find the different interferon response of bronchial epithelial cells to RV between asthmatic patients and normal subjects and the reason is unknown. By immunohistochemistry, Zhu et al.^42^ demonstrated lower interferon‐α and β expression in asthmatic bronchial biopsy in vivo at baseline and after RV infection compared to that of normal subjects. The same study also showed lower subepithelial monocytes or macrophages expressing interferon‐α and β in asthma during RV infection. The latter part was consistent with other studies showed deficient interferon‐α expression in bronchoalveolar lavage cells or alveolar macrophage infected with RV ex vivo in asthma.^13^, ^15^ Data about impaired interferon‐α production of PBMC during RV infection in asthma was conflict.^13^, ^43^, ^44^ Because pDCs is the major source of interferon‐α in PBMC,^16^, ^17^ the present study explored if the interferon‐α expression in pDCs was impaired under TLR‐7 or RV stimulation in asthma. It has been showed that the RV induced interferon‐α of pDCs was impaired under FcϵRIα cross‐linking in subset of asthmatic patients.^19^, ^20^ By purifying pDCs from patients with severe asthma, Wright et al showed numerical lower level of interferon‐α in pDCs culture (*n* = 6) after TLR‐7 stimulation without statistical difference compared to controls. Compared to the work of Wright et al, more subjects were tested in the TLR‐7 stimulation in our work (*n* = 15, Figure 5) and cells were incubated with 30% patient's own serum to simulate the original environment. Comparable with our results, Gill et al.^36^ reported the impaired production of interferon‐α from pDCs in asthma patients exposed to influenza A. Our results also revealed a significant increase in the intracellular expression of interferon‐α in pDCs after 48‐ and 96‐h exposure to RV in normal subjects but not in patients with asthma. These results suggest that the function of pDCs in asthma patients may be exhausted under prolonged RV stimulation, thereby compromising virus clearance. The functional impairment of pDCs after prolonged RV infection may also play a role in enhancing asthma‐related inflammation. One previous study on PBMCs following 5‐day RV stimulation reported an increase in Th2 inflammation under pDC depletion.^45^

The interferon‐α in the culture supernatant was not significantly different in asthma compared to normal subjects, which was not consistent with the results of intracellular staining in the present study. The discrepancy of interferon production with TLR‐7 or RV stimulation between different cell types has been reported.^13^, ^46^ These studies also showed no difference in interferon production of PBMC culture after TLR‐7 or RV stimulation in asthma or normal subjects. One possible reason for the discrepancy between interferon‐α protein concentrations in culture supernatants and intracellular interferon‐α expression determined by flow cytometry is because the ELISA experiment represents interferon‐α from all cell types in PBMCs, not only pDCs. Therefore, we are interesting how the pDCs express interferon‐α among PBMCs under stimulation. By intracellular stating in the experiment of TLR‐7 stimulation, we did find the difference of intracellular interferon‐α expression of pDCs between asthmatic subjects and normal subjects (Figure 5) and further test showed elevated interferon‐α expression under RV stimulation persisted up to 96‐h in normal subjects (Figure 6).

We observed no difference between asthmatic patients and normal subjects in the proportion of pDCs in PBMCs. This contradicts the report by Spears et al.^47^ that the number of pDCs was higher in asthma patients than in normal subjects. The difference between the two studies may be the relatively small number of patients in the present study or fact that we employed PBMCs as the denominator, whereas Spears used total leukocytes as the base.

Multiple diagnostic methods have identified RV in the upper airway of 4.5%–16% of asthmatic patients. These individuals tend to present pronounced asthma symptoms, poor lung function, and heightened sensitivity to aeroallergens.^48^, ^49^ In the current study, RV+ patients were more likely to have a history of exacerbation prompting emergency intervention. Interestingly, baseline interferon‐α levels were higher in the pDCs of RV+ patients than those of RV− patients; however, the reason for this has not been elucidated. One hypothesis is that chronic stimulation from RV in the upper airway initially induces high baseline interferon‐α levels, which blunts the response to further stimulation (e.g., TLR‐7 or prolonged infection). Further research will be required to clarify this point.

The current study has a number of limitations. First, we opted to forego the use of purified pDCs in favor of identifying pDCs in PBMCs and then incubating these cells in the patient's own serum to more closely simulate the natural course of the disease. Under these conditions, interferon‐α assays using ELISA revealed an increase in the total production of interferon‐α from PBMCs in normal subjects and asthmatic patients. Nonetheless, assessments based on this analysis would not necessarily be generalizable beyond the phenotype of the subject. Note that our results of impaired TLR‐7 and RV stimulation are in line with those obtained in previous works using different methods.^22^, ^36^ Second, the relatively small number of patients in this study precluded an exploration of the regulatory mechanisms underlying the pathogenesis of asthma. Third, the present study just observed the general effect of RV on interferon production in pDCs but did not intend to distinguish the roles of indirect activation and direct infection.^50^ We did not evaluate regulatory receptors of ITAM and MAVS‐RIGI pathway in pDCs during RVs stimulation. TLR‐3 function, at this point, it is unknown whether RVs could replicate in pDCs or whether double‐stranded RNA would promote the TLR‐3 response. This should be determined by purifying pDCs and utilizing ultraviolet‐inactivated versus live RV. However, one previous study reported that RVs do not replicate inside monocytes.^51^ Fourth, the ELISA of interferon‐α was affected by Breldin‐A in the last 3 h of experiments and represented the production from whole PBMC but not pDCs alone. However, we though the data was still worthy to present as the supporting data.

In conclusion, our findings indicate that the production of interferon‐α under TLR‐7 stimulation is compromised in the pDCs of asthmatic patients. We also found that IL‐4 can undermine the effects of TLR‐7 stimulation in normal subjects. The duration of interferon‐α expression in pDCs under RV stimulation was shortened among asthma patients than among normal subjects. Our results provide evidence that interferon‐α expression in the pDCs of asthma patients may be blunted under prolonged RV stimulation, thereby compromising RV clearance capacity.

## AUTHOR CONTRIBUTIONS

Ting‐Yu Lin and Horng‐Chyuan Lin: contributions to conception and design, acquisition of data, analysis and interpretation of data. Chun‐Yu Lo, Kuo‐Chien Tsao, and Po‐Jui Chang: been involved in drafting the manuscript and revising it critically for important intellectual content. Chih‐His Scott Kuo, Yu‐Lun Lo, and Shu‐Min Lin: revising it critically for important intellectual content and acquisition of data. Meng‐Heng Hsieh, Tsai‐Yu Wang, and Ping‐Chih Hsu: contributions to acquisition of data and interpretation of data.

## ETHICS STATEMENT

The study was approved by the Chang Gung Medical Foundation Institutional Review Board (No. 201601945A3). All subjects provided written informed consent.

## Supporting information

Supporting information.

## Data Availability

The data that support the findings of this study are available from the corresponding author upon reasonable request.
