# Supplementary material for: Impaired interferon‐α expression in plasmacytoid dendritic cells in asthma
Source: Immun Inflamm Dis. 2020 Nov 25;9(1):183–95. doi: 10.1002/iid3.376 (PMC7860612; doi:10.1002/iid3.376)
Supplement: Supplementary file 1 — Supporting information. [file IID3-9-183-s001.docx]

**Supporting information**

**Table S1. Characteristics of recruited subjects**

|  | **Normal subjects (n=26)** | **Asthmatic patients (n=26)** |
| --- | --- | --- |
| Male, n (%) | 11 (42.3) | 8 (30.8) |
| Age, year | 41.1±9.8 | 54.1±10.2*** |
| Atopy, n (%) | 2 (7.8) | 17 (65.4)*** |
| Body mass index (kg/m2) | 24.1±4.1 | 27.2±5.0* |
| Prebronchodilator FVC, liter | 2.7±0.7 | 2.6±0.9 |
| Prebronchodilator FVC, % of prediction | 92.7±17.2 | 80.6±17.4 |
| Prebronchodilator FEV1, liter | 2.3±0.6 | 1.9±0.7 |
| Prebronchodilator FEV1, % of prediction | 93.0±14.7 | 73.8±19.4* |
| FEV1/FVC, % | 85.5±2.5 | 82.8±15.3 |
| Treatment with inhaled corticosteroid and long-acting bronchodilators, n | 0 | 23 (88.5)*** |
| Treatment with oral corticosteroid, n, (%) | 0 | 11 (42.3)*** |
| Treatment with biological agent, n, (%) | 0 | 6 (23.1)* |

Data are expressed as means ± SEM or number of subjects with percentage (*p<.05, **p<.01, ***p<.001)

Definition of abbreviations: FVC= forced vital capacity; FEV1= forced expiratory volume in one second


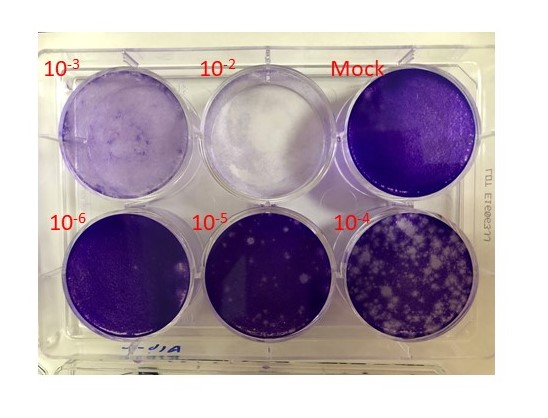


**Figure S1. Virus titers of RV-A16 were determined by plaque assay.** Confluent H1-Hela cells in 6-well plates were infected with the virus stock serially diluted from 10^-2^ to 10^-6^. The virus titer of RV-A16 passage 2 was 1×10^7^ pfu/ml, and an MOI (multiplicity of infection) of 1 WA used in subsequent experiments.


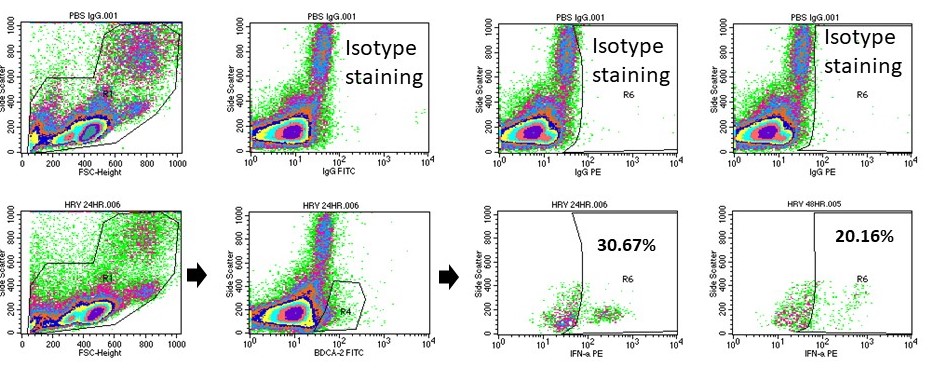


**Figure S2. Gating strategy for intracellular interferon-α staining of pDCs under RV stimulation.** PBMCs were stimulated with RV. BDCA-2(+) pDCs gated from PBMCs (left two panels) and interferon-α-PE gated from BDCA-2 following RV stimulation for 24 and 48 hours (right two panels).

**Figure S3.** **Interferon-α levels in the supernatant of PBMC cultures from asthmatic patients or normal subjects under the following conditions:** (A) Culture treated with PBS for 6 hours, (B) after TLR-7 stimulation using Gardiquimod (GDQ) for 6 hours, (C) after TLR-7 stimulation for 6 hours and IL-4 pretreatment for 1 hour, and (D) TLR-7 stimulation for 6 hours and IL-13 pretreatment for 1 hour.

**Figure S4. Expression of intracellular interferon-α in pDCs after Gardiquimod stimulation as a function of disease severity and status of atopy.** pDCs of (A) mild-to-moderate asthmatics, (B) severe asthmatics, (C) atopic asthmatics, and (D) non-atopic asthmatics. GDQ: Gardiquimod.


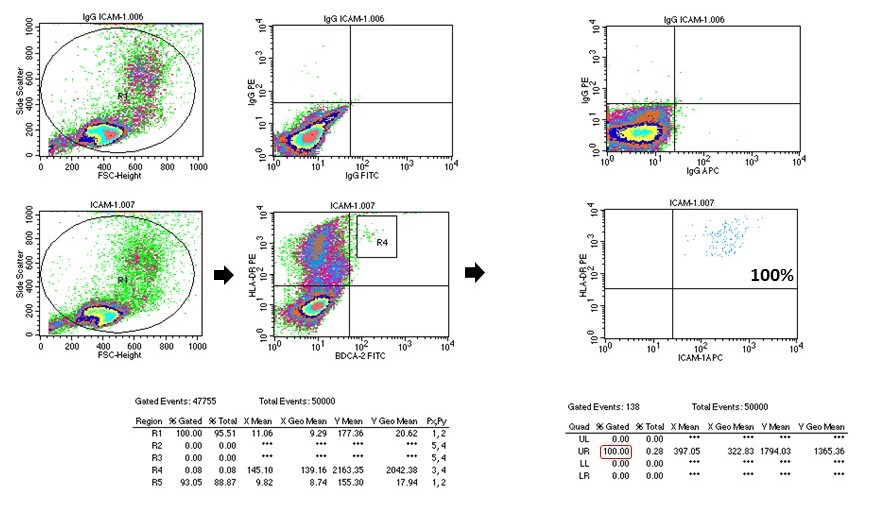
**Figure S5. Expression of ICAM-1 on BDCA-2(+) and HLA-DR (+) pDCs**

**Figure S6. Production of interferon-α in the PBMCs (left panel) and intracellular interferon-α expression of pDCs (right panel) of asthmatic patients and normal subjects** (A) after TLR-7 agonist, Gardiquimod (GDQ), (B) stimulation, with rhinovirus (RV) for 24 hours and (C). stimulation with rhinovirus for 48 hours.

**Figure S7. Interferon-α production by PBMC culture after 24-hour and 96-hour RV stimulation with or without IL-4 pretreatment.** (A) Normal subjects and (B) asthmatic patients.

**Figure S8. Associations between baseline intracellular interferon-α in pDCs and (A) FVC % of prediction, (B) FEV1 % of prediction, (C) FEV1/FVC %, (D) post bronchodilator FEV1 change, (E) annular FEV1 change (%), and (F) annular FEV1 change (ml). Baseline expression of intracellular interferon-α in pDCs of (G) atopic asthmatic vs. non-atopic asthmatic and (H) mild-to-moderate asthmatic vs. severe asthmatic**

**Figure S9. Expression of intracellular interferon-α in asthmatic patients identified as RV+ and without RV identification under various conditions:** (A) treatment with PBS, (B) TLR-7 stimulation, and (C) TLR-7 stimulation with pretreatment of IL-4.
